# Medical Feature Extraction From Clinical Examination Notes: Development and Evaluation of a Two-Phase Large Language Model Framework

**DOI:** 10.2196/78432

**Published:** 2025-12-03

**Authors:** Manal Abumelha, Abdullah AL-Malaise AL-Ghamdi, Ayman Fayoumi, Mahmoud Ragab

**Affiliations:** 1 Information Systems Department Faculty of Computing and Information Technology King Abdulaziz University Jeddah Saudi Arabia; 2 Information Systems Department Applied College at Khamis Mushait King Khalid University Abha Saudi Arabia; 3 Computer Science Department School of Engineering, Computing and Design Dar Alhekma University Jeddah Saudi Arabia; 4 Information Technology Department Faculty of Computing and Information Technology King Abdulaziz University Jeddah Saudi Arabia

**Keywords:** automated medical assessment, clinical NLP, hallucination mitigation, instruction tuning, large language models, medical feature extraction, semantic matching

## Abstract

**Background:**

Medical feature extraction from clinical text is challenging because of limited data availability, variability in medical terminology, and the critical need for trustworthy outputs. Large language models (LLMs) offer promising capabilities but face critical challenges with hallucination.

**Objective:**

This study aims to develop a robust framework for medical feature extraction that enhances accuracy by minimizing the risk of hallucination, even with limited training data.

**Methods:**

We developed a two-phase training approach. Phase 1 used instructing fine-tuning to teach feature extraction. Phase 2 introduced confidence-regularization fine-tuning with loss functions penalizing overconfident incorrect predictions, which were captured using bidirectional matching targeting hallucination and missing features. The model was trained using the full data of 700 patient notes and on few-shot 100 patient notes. We evaluated the framework on the United States Medical Licensing Examination Step-2 Clinical Skills dataset, testing on a public split of 200 patient notes and a private split of 1839 patient notes. Performance was assessed using precision, recall, and *F*_1_-scores, with error analysis conducted on predicted features from the private test set.

**Results:**

The framework achieved an *F*_1_-score of 0.968-0.983 on the full dataset of 700 patient notes and 0.960-0.973 with a few-shot subset of 100 of 700 patient notes (14.2%), outperforming INCITE (intelligent clinical text evaluator; *F*_1_=0.883) and DeBERTa (decoding-enhanced bidirectional encoder representations from transformers with disentangled attention; *F*_1_=0.958). It reduced hallucinations by 89.9% (from 3081 to 311 features) and missing features by 88.9% (from 6376 to 708) on the private dataset compared with the baseline LLM with few-shot in-context learning. Calibration evaluation on few-shot training (100 patient notes) showed that the expected calibration error increased from 0.060 to 0.147, whereas the Brier score improved from 0.087 to 0.036. Notably, the average model confidence remained stable at 0.84 (SD 0.003) despite *F*_1_ improvements from 0.819 to 0.986.

**Conclusions:**

Our two-phase LLM framework successfully addresses critical challenges in automated medical feature extraction, achieving state-of-the-art performance while reducing hallucination and missing features. The framework’s ability to achieve high performance with minimal training data (*F*_1_=0.960-0.973 with 100 samples) demonstrates strong generalization capabilities essential for resource-constrained settings in medical education. While traditional calibration metrics show misalignment, the practical benefits of confidence injection led to reduced errors, and inference-time filtering provided reliable outputs suitable for automated clinical assessment applications.

## Introduction

### Overview

Medical documentation is a critical practice in clinical systems, requiring practitioners to document patient history, symptoms, and potential diagnoses. This documentation is evaluated through standardized medical examinations such as the United States Medical Licensing Examination (USMLE) [1]. These evaluations are essential for assessing whether students can effectively align clinical knowledge with professional communication skills [2]. The Step 2 Clinical Skills examination evaluates the students’ ability to recall relevant knowledge for a specific case and document key features contributing to an accurate diagnosis [3,4].

The high resource requirements of these evaluations create substantial difficulties for educational institutions. More than 30,000 medical notes need evaluation each year, which requires 100 physicians to dedicate substantial time to extensive manual assessment [3]. These evaluation methods create resource challenges, along with requirements of consistency, reliability, and timely feedback among different examination centers in different locations [5,6]. Recent studies highlight the potential of large language models (LLMs) to address these challenges by automating clinical note assessment with high accuracy and scalability [7,8]. These concerns highlight the need for efficient and reliable assessment tools applicable everywhere with consistent performance [9].

Automation of this process began early through statistical natural language processing (NLP) approaches that relied on n-gram models and fuzzy logic. These approaches used character-based statistics with limited capability in capturing the contextual and semantic information in medical text [3,4,10]. Recent bidirectional encoder representations from transformers (BERT)–based approaches have demonstrated better semantic understanding for medical document assessment but still exhibit weaknesses in broader application areas and need intensive span-level annotation of sentences to reach effective training capabilities [5,6,9].

LLMs have revolutionized NLP functionalities across all sectors [11]. These models display impressive capabilities to learn from zero-shot and a few-shot inputs, which could help address data shortage problems affecting medical education settings [12]. Medical organizations need to address interpretability and hallucination when using LLMs to extract valuable features in high-risk scenarios [13-15].

Confidence calibration aims to reduce the gap between the model’s predicted confidence score and its actual performance evaluation, especially in health care applications and educational assessment [16]. Traditional confidence calibration is effective for classification [17-19], but challenges persist in deep neural networks as well as pretrained language models [16,20]. For earlier neural networks, integrating noise or penalties into the cross-entropy loss can improve generalization and reduce overconfident errors [21,22]. The ability to calibrate the internal confidence of generative LLMs remains challenging and is an active research area, as these models are pretrained to maximize the likelihood of sequential output [23].

### Related Work

The field of automated medical note assessment has expanded to address the high costs, human bias, and scalability issues of manual reviews. Early work relied on statistical and rule-based techniques. Latifi et al [10] tested decision tree models to evaluate student examination responses, whereas Yim et al [4] developed scoring methods based on n-gram features with lexical matching in note assessment. Applying these approaches brought only restricted context comprehension and an inability to manage clinical language inconsistencies. The INCITE (intelligent clinical text evaluator) system [3] introduced semisupervised techniques, incorporating fuzzy matching through Levenshtein distance and lexicon-based processing. Although these methods retrieved more data, they required near-exact string matches and failed to generalize semantics, particularly for complex or paraphrased symptom descriptions. The field progressed with transformer-based models. Ganesh and Bansal [24] proposed a transformer-based method for automatically mapping clinical notes to specific clinical concepts. Zhou et al [5] used a BERT model trained with their own data to spot key features, even with limited supervision. Xu et al [6] applied DeBERTa (decoding-enhanced bidirectional encoder representations from transformers with disentangled attention), integrating pseudo-labeling and masked language modeling. Tan et al [25] ensembled DeBERTa transformer models using multitask learning for both note-based and character-based approaches to automatically score USMLE patient notes. Yaneva et al [9] integrated transformer models with rule-based matching syntax, which made their system excel in automating the USMLE Step-2 Clinical Skills assessment.

Despite these advances, such models demand extensive annotated data and lack the semantic depth and contextual reasoning offered by autoregressive LLMs. Vithanage et al [26] demonstrated LLMs’ superior ability to extract structured information from medical reports. Their deep contextual understanding has proven successful across many clinical applications [27,28]. However, challenges remain, including high computational demands and hallucination risks that threaten reliability [13,27].

Research on hallucination detection and mitigation has become critical, with uncertainty and confidence estimation methods showing promise [29,30]. Confidence calibration seeks to align a model’s predicted confidence with its actual performance. Traditional calibration suits classification tasks, in which each class has a distinct probability, but generative LLMs—trained to maximize confidence across entire sequences—often exhibit overconfidence [16]. Xiao and Wang [31] used predictive uncertainty scores to detect hallucinations, modifying beam search at inference time as a post hoc mitigation method, distinct from training-time regularization. The character n-gram *F*_1_-score (chrF), introduced by Popović [32] for machine translation evaluation, captures morphological variations better than word-level metrics. Li et al [33] used a Dice loss *F*_1_-score to narrow the gap between the model performance and final evaluation *F*_1_-scores. In machine translation, chrF correlates strongly with human judgments, but its potential as a training objective for sequence models remains underexplored. Our work builds on this by incorporating chrF scores as a regularization signal in the loss function.

We use confidence scores to penalize hallucinations and missing features during training to improve *F*_1_-scores in medical feature extraction. This approach encouraged the model to be more conservative when overconfident with incorrect outputs, mitigating LLMs’ inherent overconfidence [34,35]. Key literature gaps include traditional methods’ substantial difficulties when confronted with the inherent semantic variability characteristic of clinical terminology [9,10], LLMs’ data scarcity and the persistent risk of hallucination [13-15], and manual assessment’s scalability limitations [1,3,4]. Our two-phase LLM framework uses instructing fine-tuning to teach extraction, followed by confidence-regularization fine-tuning by modifying the cross-entropy loss function with feature-based confidence aligned with *F*_1_ performance to reduce hallucinations and enhance feature capture, thereby improving accuracy and generalization in low-resource settings.

### Study Aim and Contributions

Our framework adopts confidence-regularization fine-tuning to penalize hallucinated and missing features, incorporating this as a training signal in the LLM, to enhance medical feature extraction.

We introduce an end-to-end framework that integrates the instruction-understanding capability of an LLM with confidence regularization, used as a signal to avoid hallucinations and missing features even with limited resources. This work offers several key contributions, beginning with introduction of an end-to-end framework that combines instructing fine-tuning power with model confidence-regularization fine-tuning, along with semantic embedding matching, to extract medical features from patient notes. Additionally, we designed a bidirectional matching mechanism that evaluates the extracted features by identifying hallucinated and missed features, enabling precise feedback and structured adjustment during training. To achieve this we developed a multipenalty learning objective that uses model confidence as a regularization signal to improve the reliability of medical NLP predictions.

In evaluations on USMLE Step-2 Clinical Skills notes, our framework achieved state-of-the-art performance with *F*_1_=0.983 (semantic matching) and *F*_1_=0.968 (binary overlap) on the entire dataset and maintained high performance with *F*_1_=0.973 (semantic matching) and *F*_1_=0.952 (binary overlap) while using only 100 of 700 patient notes, representing 14.2% of the training data. Furthermore, it reduced hallucinated outputs by 89.9% (from 3081 to 311 hallucinated features) and increased feature detection by 88.9% (from 6376 to 708 missing features) in the final model compared with the base vanilla model (Mistral Nemo with few-shot in-context learning [ICL]), thereby paving the way for reliable medical NLP under limited supervision. The aim of this study was to develop and evaluate a two-phase LLM framework for automated medical feature extraction from clinical examination notes, achieving high accuracy with minimal training data while reducing hallucination and missing feature errors to support reliable medical education assessment.

## Methods

### Study Overview and Research Questions

Our framework consists of a two-phase training approach: instructing fine-tuning and confidence-regularization fine-tuning. Before explaining each phase, we need to define the task. In the medical feature extraction task, given a medical history text p belonging to P and a set of target features to find F = {*f*_1_*, f*_2_*, … ,f*_n_}, the goal is to extract text segment semantically equivalent to features E = {*e*_1_*, e*_2_*, … , e*_n_} from P. If no corresponding segment exists, then *e*_i_=Ø.

These questions guide our experimental design to comprehensively evaluate the proposed framework: (1) research question (RQ) 1: Is the model able to generalize effectively from limited training data, while maintaining competitive performance against full-data training? (2) RQ2: Does the proposed framework enhance performance by reducing hallucination and increasing the ability to extract clinical features? (3) RQ3: Does the incorporated confidence-regularization strategy enhance the alignment between the model’s confidence scores and its true extraction performance?

### Dataset and Experimental Design

**Figure 1:**
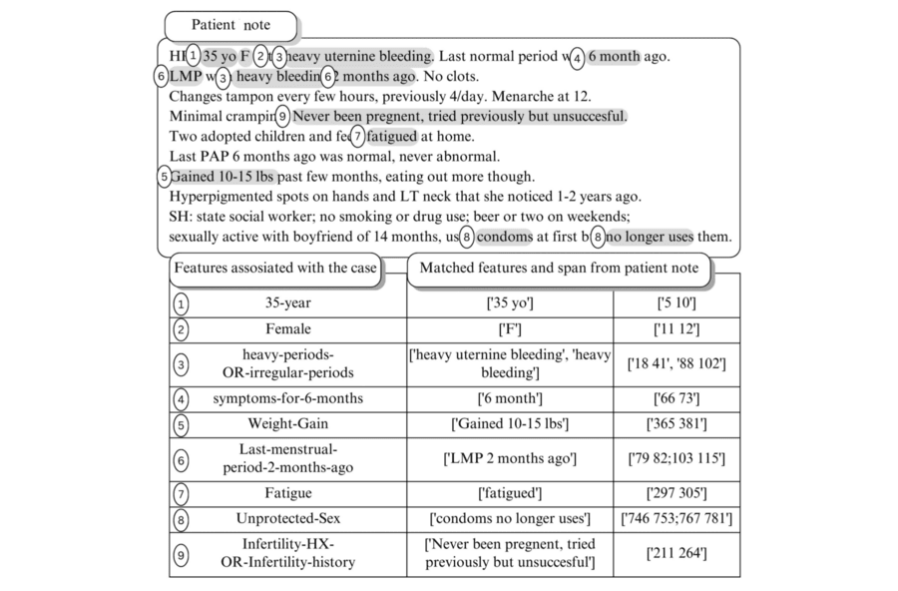
Sample of patient note from the United States Medical Licensing Examination (USMLE) Step-2 Clinical Skills dataset, showing case-associated features (to be identified) and annotated features (already identified by experts) [38].

The research collected data from 2017 to 2020 from examination sites across the United States, obtaining data from 35,156 medical students [36]. The dataset includes 10 clinical case scenarios represented across 43,985 patient notes. The training data comprised 2840 patient notes, equally distributed at 284 per clinical case, as illustrated in Figure 1.

The dataset annotation task was performed by 10 expert annotators working in 5 pairs. A double-annotation process was applied to 29 notes per case to compute *F*_1_ agreement scores, defining each case’s feature spans. The interannotator agreement, measured through *F*_1_, was 84%, while binary detection of features reached 97% agreement. The full annotation process is detailed elsewhere [36].

The available dataset exists in two splits: a public split and a private split. The public split included 1000 of the 2839 records made available through the Kaggle competition [37], with each case containing 100 notes. The private split was used to evaluate the submitted notebook in the competition and consists of 1893 records. For fair comparison, we followed the same split as the competition and previous study by Yaneva et al [9]. Table 1 illustrates the data split used in this experiment.

**Table 1:** Dataset split showing the number of annotated patient notes and features used for training. There is no overlap between data splits.

Dataset split	Dataset size	Annotated features
Training data	700 patient notes	10,011 features
Validation data	100 patient notes	1430 features
Test data (public split)	200 patient notes	2860 features
Test data (private split)	1839 patient notes	20,360 features
Total annotated data	2839 patient notes	34,661 features

### Two-Phase Training Framework

#### Phase 1: Instructing Fine-Tuning

Recent developments have enabled language models to follow human natural language instructions [39]. In contrast, earlier models required task-specific supervised fine-tuning on labeled data, which demanded intensive effort to prepare data for task-specific requirements [38].

In this study, we use instructing fine-tuning, in which a LLM is trained on pairs of instructions and responses [40]. The first phase of our methodology involves fine-tuning a pretrained language model on medical feature extraction tasks. Using Mistral Nemo instruct [41] as our backbone model θ_0_. Mistral Nemo was chosen as the backbone for this model, based on our previous experiment with few-shot learning on different open-source models, where Nemo showed the best performance among them [42]. We then fine-tuned Mistral Nemo on a dataset 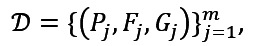 where P_j_ is patient history, F_j_ is a set of target features, and G_j_ is the corresponding set of ground truth extractions for that specific patient history.

LLM predictions can vary significantly based on the design and quality of prompt templates [43]. We carefully designed our instruction template to be optimized for feature extraction tasks.

The model was trained to autoregressively generate output tokens based on the input prompt. The training objective was to minimize the negative log-likelihood loss between the generated tokens and the outputtokens across all training examples.

#### Phase 2: Confidence-Regularization Fine-Tuning

**Figure 2:**
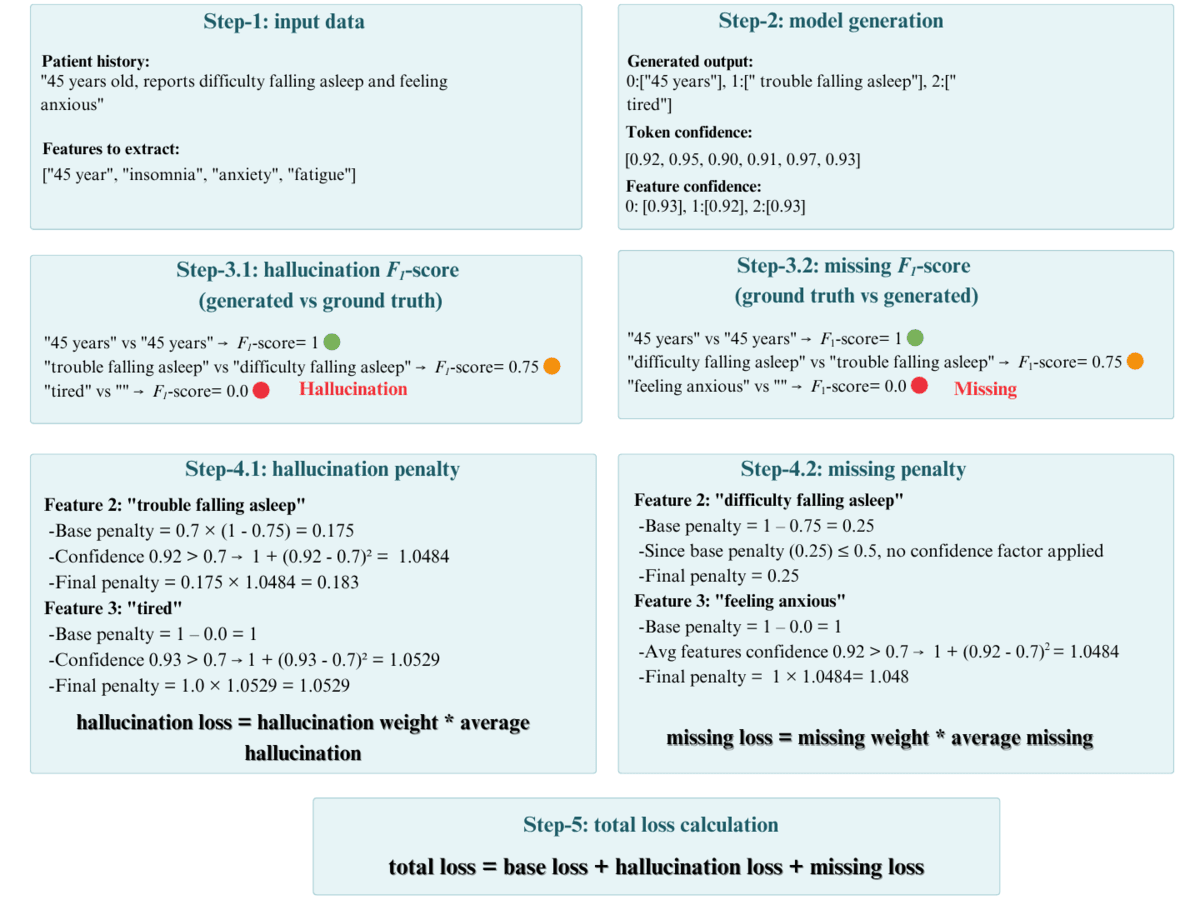
Five steps of confidence-regularization fine-tuning phase illustrated using the process flow of one example.

The first fine-tuning step instructs the model to perform feature extraction tasks, enabling it to extract features that can later be refined by penalizing poor performance. Confidence calibration is used to align the model’s confidence scores with its actual performance. The complex architecture of deep neural networks, with an increased number of layers, allows them to handle larger datasets [44]. However, this complexity poses a calibration challenge, as such models are trained to maximize the probability of predicted outputs, leading to inherently overconfident predictions. We achieve this through a customized loss function that penalizes overconfident incorrect predictions, drawing on previous work in uncertainty estimation [22,45]. Unlike post hoc methods such as temperature scaling [16] or external confidence mapping [45], our approach integrates regularization directly into training, ensuring robust feature extraction. The confidence-regularization fine-tuning process is illustrated in Figure 2, with an example detailing the process for a single training instance as outlined in the following steps.

- Step 1–input data: The data used as input in the model for the second training phase consists of patient notes and features to look for, without including the ground truth, because the model has already been trained to extract features properly. The ground truth is used in the subsequent steps.
- Step 2–model generation: The model extracts features, and the confidence of these features is obtained using token-level probabilities from the softmax layer [45,46]. Modern LLMs tend to be overconfident, especially if they are already pretrained using supervised pretraining [47]. To overcome this limitation, and inspired by previous work, we made 3 adjustments to the confidence score to make it less flat and more varied. First, we applied length normalization to boost confidence scores for shorter outputs and reduce them for longer ones, correcting biases as shown by Murray and Chiang [48] and Farquhar et al [46]. Second, inspired by Kuhn et al [49], who used bidirectional entailment to group semantically similar outputs and calculate semantic entropy for better uncertainty estimation, and Vazhentsev et al [50], who examined averaging token probabilities and entropy, we averaged token-level probabilities per feature to generate more robust feature-based confidence scores, facilitating effective penalization in our model. Third, Gal et al [51] injected Gaussian noise into logits during training to regularize and prevent model overconfidence. Similarly, Li et al [33] added noise to confidence outputs before generating pseudo-labels to improve calibration. Inspired by them, we injected controlled stochastic randomness to prevent the confidence scores from collapsing into a narrow range of confidence values.
- Step 3–bidirectional semantic mapping: We targeted two model behaviors: hallucination and missing features. Hallucination refers to extracting nonexistent features from patient notes and is measured by forward-matching predicted and ground truth features, using an n-gram *F*_1_-score to give credit for partial matches. This matching is performed as follows: for each generated feature, we calculate its *F*_1_ similarity score against all ground truth features and select the highest score. Features are penalized based on their best *F*_1_ match: those with *F*_1_>0.3 receive a reduced penalty of 0.7 × (1 – *F*_1_), while those with *F*_1_ ≤0.3 receive the full penalty of (1 –*F*_1_). For missing feature detection, we perform the reverse process: each ground truth feature is matched against all generated features to find its highest *F*_1_ -score. Ground truth features that are poorly matched (<0.5) indicate missing extractions and contribute to the missing feature penalty, calculated as 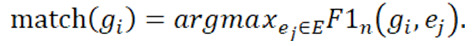.
- Step 4–calculate penalties: Drawing on previous work, such as Zou et al [52], who penalized overconfident incorrect predictions during model training; Nan et al [53], who introduced entity overlap metrics to promote faithful information extraction; and Liang et al [18], who incorporated a penalty term based on the disparity between predicted confidence and accuracy to enhance calibration, we propose a novel approach that integrates *F*_1_ evaluation metrics into our penalty term. Specifically, we introduced an *F*_1_-based confidence penalty that scales with the confidence of incorrect extractions, penalizing them proportionally to their predicted confidence to improve model calibration and extraction accuracy. Penalties are calculated only for overconfident incorrect predictions compared with the confidence threshold. Thus, backpropagation occurs exclusively for overconfident, incorrect predictions; if there is no penalty, no gradient is propagated. The *F*_1_-based confidence penalty extends the Brier score’s calibration framework by measuring performance through (1 − *F*_1_) and penalizing overconfident predictions with a quadratic term when confidence exceeds a threshold. The scaling factor α is set to 2 to increase the penalty’s sensitivity to overconfidence. For confidence-adaptive training and stabilized training, we apply a confidence feedback loop to prevent training instability by smoothing confidence estimates across epochs and a dynamic threshold to create curriculum-like learning by increasing the threshold gradually. Without these modifications, the model becomes sensitive to the penalty and collapses during training, by generating redundant patterns.
- Step 5–total loss calculation: Our training approach combines multiple objectives to enhance the model’s ability to extract accurate and complete features. We start with a standard language modeling cross-entropy loss to predict correct tokens, then add a hallucination penalty, calculated using *F*_1_-based metrics, to discourage overconfident incorrect predictions by scaling penalties with confidence scores. To address missing features, a complementary penalty reduces the confidence when features are missing, using a backward-mapping mechanism. The missing feature penalty is weighted higher (0.5) than the hallucination penalty (0.2), based on the model’s previous result from the first training phase, where recall was less than precision.

The confidence-regularization fine-tuning process improves deep neural network performance by penalizing overconfident, incorrect predictions. It begins with inputting patient notes for feature extraction, followed by generating token-level confidence scores through the softmax layer, adjusted through length normalization, averaged token probabilities, and injected stochasticity to reduce overconfidence. Bidirectional semantic matching addresses hallucination and missing features by computing forward and backward *F*_1_-scores to align predicted and ground truth features, ensuring comprehensive error detection. A novel *F*_1_-based confidence penalty, inspired by previous work, scales penalties for overconfident incorrect predictions using a quadratic term and a dynamic threshold (τ from 0.6 to 0.7) for curriculum learning, promoting stable convergence. The total loss integrates cross-entropy loss with weighted hallucination (0.2) and missing feature (0.5) penalties, thereby prioritizing recall improvement. No backpropagation occurs for cases where no penalty is incurred. Heuristic values for thresholds and variables were selected to balance the penalty and training stability, preventing model collapse due to redundant generation at the middle of the first epoch. Detailed formulas and descriptions are provided in Multimedia Appendix 1.

As detailed in Algorithm 1 in Multimedia Appendix 2, our framework implements two-phase training to enhance medical feature extraction. First phase, an instructing fine-tuned model generates feature and confidence scores, adjusted by a complexity factor and a dynamic threshold based on the prior epoch’s *F*_1_*-*score. Second phase, a confidence-regularization fine-tuning based on bidirectional matching identifies hallucinated and missing features, overconfidence with low *F*_1_-score are penalties and calculated, alongside a base loss. This dual-objective strategy refines predictions by penalizing the hallucination and missing features behavior.

### Implementation Details

We conducted two training phases. The first phase, instructing fine-tuning, aims to enable the LLM to perform the information extraction task by providing detailed instructions and input-output pairs; this phase aligns the model with the extraction task and uses Mistral Nemo [37] as the base model. The second phase, confidence-regularization fine-tuning, aims to reduce hallucination and minimize missing feature extraction, thereby increasing the performance; this phase uses the model from the instructing fine-tuning phase as the base model. The confidence-regularization phase uses *F*_1_-score n-gram matching to accurately evaluate the model’s extraction compared with the ground truth features annotated by experts and compares them against the confidence of the predicted features.

For comparison, we trained two variants of each phase: one using a limited dataset (100 patient notes) with 90 patient notes held out for validation to evaluate LLM performance in low-resource settings, and another using the full training corpus (700 patient notes) with 100 patient notes held out for validation. The following are the key hyperparameters used in both training phases. During instructing fine-tuning, we use the Mistral Nemo instruct model with a batch size of 4, learning rate of 5e−5, weight decay of 0.01, and dropout set to 0.1. Optimization was performed using AdamW (PyTorch, Meta AI) with a linear learning rate scheduler across 5 epochs, with early stopping based on the validation *F*_1_-score. For confidence-regularization fine-tuning, we retained the same optimization setup (batch size, learning rate of 1e−4, dropout, weight decay, and epochs) but introduced confidence-regularization parameters: α=0.7 as the feedback coefficient, β=0.2 as the stochastic scaling factor, and λ=20 as the feature complexity normalizer. The dynamic confidence threshold is initialized at τ*_init_*=0.6 and gradually increases to τ*_final_*=0.7 during training. In the final configuration, we assign loss weights of 0.2 for hallucinated features and 0.5 for missing features. All experiments are conducted on NVIDIA H100 GPUs (graphics processing unit; 80 GB; NVIDIA corporation). These hyperparameters reflect the configurations reported in our Tables S1-S4 in Multimedia Appendix 2.

### Evaluation Framework

#### Performance Evaluation

During inference, the trained model extracts features from new patient notes using few-shot ICL. Each note is prompted with standardized instructions and 4-6 exemplary input-output pairs to guide feature generation. Extracted features undergo a three-step validation process, referred to as the matching gate: (1) exact matching with regular expressions and Levenshtein distance for misspellings, (2) nonconsecutive and sentence-level matching using term frequency-inverse document frequency similarity and segment alignment, and (3) overlap and windowed matching for distributed features. This ensures that only text-supported features are validated, preventing hallucinations [42]. To ensure fair comparison, this matching gate validation pipeline was applied consistently across all models during inference, including the vanilla baseline (Mistral Nemo with few-shot ICL), the instructing fine-tuned models, and the confidence-regularization fine-tuned models. Following Yaneva et al [9], we used a binary evaluation protocol in which features are scored as present or absent, regardless of exact span extraction. The binary evaluation proposed by Yaneva et al [9] relies on model probability for a token, which is inapplicable for a generative model; therefore, we conducted two types of binary evaluations. First, binary overlap occurs when feature words overlap with the predicted feature, with 50% considered as found. This binary decision is used to calculate (binary overlap) precision, recall, and *F*_1_ metrics. Second, to account for the LLM’s ability to capture meaning, we also implemented semantic binary evaluation. During inference, the trained model attempts to extract each target feature from the case-specific feature list. For each target feature, the system: (1) searches for matching text in the patient note using the matching gate validation (confirming existence in the patient note); (2) if found, calculates cosine similarity between the extracted text and the target feature description using a lightweight sentence embedding model [54]; (3) marks the feature as present only if cosine similarity ≥0.5; and (4) logs the evidence span with character offsets. This two-step validation process, followed by similarity matching, determines the binary presence used to calculate (semantic matching) precision, recall, and *F*_1_ metrics.

For human oversight, the system flags borderline matches (cosine similarity 0.5-0.7) and missing features for manual verification, enabling instructors to efficiently validate the automated scoring. The complete extraction metadata supports graduated review based on institutional requirements. Cosine similarity is not equivalent to the model’s internal confidence. Our calibration estimate is derived from the LLM’s softmax outputs, which serve as regularization during training to penalize overconfidence. By contrast, cosine similarity is a deterministic, embedding-based measure of semantic closeness between the extracted text and the rubric feature description, and it is an inference step to highlight borderline cases of semantic mismatch.

#### Generalization Assessment

For a comprehensive evaluation of our framework’s performance under different training settings, we trained four models using the following configurations: (1) few-shot (instructing): trained the model using instructing fine-tuning approach on 100 annotated patient notes—this training used Mistral Nemo as base model; (2) few-shot (confidence-regularization): trained the model using confidence-regularization fine-tuning approach on 100 annotated patient notes—this training used a fine-tuned model from the instructing phase as the base model; (3) full (instructing): trained the model using an instructing fine-tuning approach on 700 annotated patient notes—this training used Mistral Nemo as a base model; and (4) full (confidence-regularization): trained the model using confidence-regularization fine-tuning approach on 700 annotated patient notes—this training used a fine-tuned model from the instructing phase as the base model.

#### Hallucination and Missing Feature Quantification

The model also generates a confidence score of *c_i_* ∈ (0-1) for each extracted text segment *e_i_,* indicating the model’s estimated likelihood that *e_i_* and *f_i_* semantically match. The ground truth annotated by experts for each *f_i_* is denoted as *g_i_*, which may be Ø if no corresponding feature exists in P.

We use a character-level n-gram *F*_1_-score, denoted as *F*_1_-socre_n_(*e_i_,g_i_*), to measure the similarity between an extraction *e_i_* and the ground truth *g_i_*. Compared with exact string matching, this character-level method better captures partial matches and variations in medical terminology. The *F*_1_-score is calculated as:



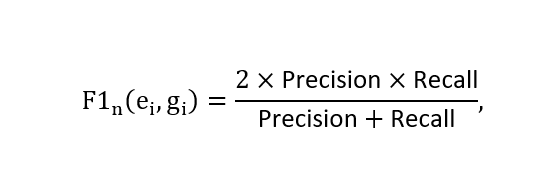



Where:



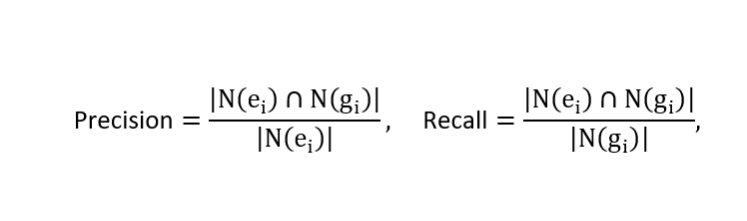



N (x) represents the set of character n-grams in text x.

For all models evaluated in this study, including the vanilla baseline (Mistral Nemo with few-shot ICL), instructing fine-tuned models, and confidence-regularization fine-tuned models, the same inference pipeline was applied. This pipeline includes few-shot ICL and the three-step matching gate validation described in the Performance Evaluation section, ensuring fair comparison of hallucination and missing feature rates across all model variants.

### Calibration Analysis

To assess the alignment between the model’s predicted confidence and its actual performance, we used two standard calibration metrics: the expected calibration error (ECE) and the Brier score. ECE measures the average difference between the model’s confidence scores and its actual accuracy across predefined confidence bins, providing insight into systematic overconfidence or underconfidence. The Brier score quantifies the mean squared difference between predicted confidence and true outcomes, reflecting both calibration and predictive accuracy. Unlike the binary presence/absence evaluation used for model comparison, our calibration analysis uses continuous *F*_1_-scores calculated at the feature level. For each extracted feature, we compute chrF scores against ground truth, providing a continuous measure (0,1) that better captures partial matches and extraction quality. This continuous evaluation is more suitable for calibration assessment, as it provides granular confidence–performance alignment with actual performance rather than binary outcomes.

### Ethical Considerations

This study used the USMLE Step-2 Clinical Skills dataset, comprising deidentified patient notes from standardized medical examinations. The original data collection by the National Board of Medical Examiners (NBME) was conducted with appropriate informed consent from participating medical students who took the USMLE Step-2 Clinical Skills examination [36]. All data were fully anonymized, ensuring no identifiable information was included, and privacy was protected through NBME’s data-sharing protocols. Our data request was approved by NBME (ID 34890198). No compensation was provided, as the study did not involve direct participant recruitment. No images of individual participants were included in the manuscript or supplementary materials, ensuring no identification risks. The proposed framework is designed for research purposes, specifically for evaluating automated medical informatics and information extraction in medical education assessment, and is not intended for diagnostic use.

## Results

### Overview

We present experimental results evaluating the proposed framework on the USMLE Step-2 Clinical Skills dataset in few-shot and full-data settings, comparing it against previous state-of-the-art models [9]. The results address 3 RQs; each subsection below corresponds to one aspect, providing a clear, stepwise assessment of our two-phase framework.

### Overall Performance

Each evaluation was performed using the two splits (the public and the private) to accommodate direct comparisons with previous work. Performance across these datasets is summarized in Table 2 using precision, recall, and *F*_1_ metrics as evaluation criteria. These results demonstrate the ability of LLMs to generalize even with few-shot training. The binary evaluation relies on exact text comparison with a 50% overlap threshold, while the semantic test assesses semantic meaning match, reflecting the LLM’s ability to capture deep meaning. The results of the semantic binary evaluation outperform overlap for two reasons: (1) the semantic assessment is less strict in capturing exact matches and (2) the semantic test runs between predicted features and case-related features if there is no annotated ground truth. We examined 1430 features and found 68 false positives, as they were not annotated in the ground truth but were semantically equivalent to case-related features. The few-shot confidence-regularization model outperforms previous systems on both public and private splits, using binary evaluation [9]. The full data training model becomes state-of-the-art performance with an *F*_1_-score of 0.983, validating the effectiveness of our two-phase fine-tuning framework (instructing + confidence-regularization).

**Table 2:** Performance comparison on public and private dataset splits. Baseline models use token-level probability thresholds. Our models show results for both binary overlap (token overlap ≥0.5) and semantic matching (cosine similarity ≥0.5) approaches.

Model		Public dataset			Private dataset		
		Precision	Recall	*F*_1_-score	Precision	Recall	*F*_1_-score
**Baseline models (Yaneva et al [9])**							
	INCITE^a^	0.962	0.818	0.883	0.961	0.828	0.888
	DeBERTa^b^	0.950	0.962	0.956	0.951	0.963	0.957
	DeBERTa + MTL^c^	0.947	0.961	0.954	0.953	0.963	0.958
	DeBERTa + MLM^d^	0.952	0.961	0.957	0.961	0.956	0.958
**Our models with few-shot training (100 patient notes)–semantic match**							
	Instructing fine-tuning	0.970	0.928	0.949	0.971	0.934	0.952
	Confidence-regularization fine-tuning	0.986^e^	0.960	0.973^e^	0.982^e^	0.964	0.973^e^
**Our models with few-shot training (100 patient notes)–binary overlap**							
	Instructing fine-tuning	0.935	0.917	0.926	0.937	0.947	0.942
	Confidence-regularization fine-tuning	0.940	0.965^e^	0.952	0.950	0.971	0.960
**Our models with full data training (700 patient notes)–semantic match**							
	Instructing fine-tuning	0.979	0.952	0.965	0.974	0.952	0.963
	Confidence-regularization fine-tuning	0.988^f^	0.978^f^	0.983^f^	0.988^f^	0.974^f^	0.981^f^
**Our models with full data training (700 patient notes)–binary overlap**							
	Instructing fine-tuning	0.915	0.958	0.936	0.939	0.969	0.954
	Confidence-regularization fine-tuning	0.956	0.980	0.968	0.962	0.984^e^	0.973^e^

^a^INCITE: intelligent clinical text evaluator.

^b^DeBERTa: decoding-enhanced bidirectional encoder representations from transformers with disentangled attention.

^c^MTL: multitask learning.

^d^MLM: masked language modeling.

^e^Second best result.

^f^Best result.

### Few-Shot Learning Capability (RQ1)

**Figure 3:**
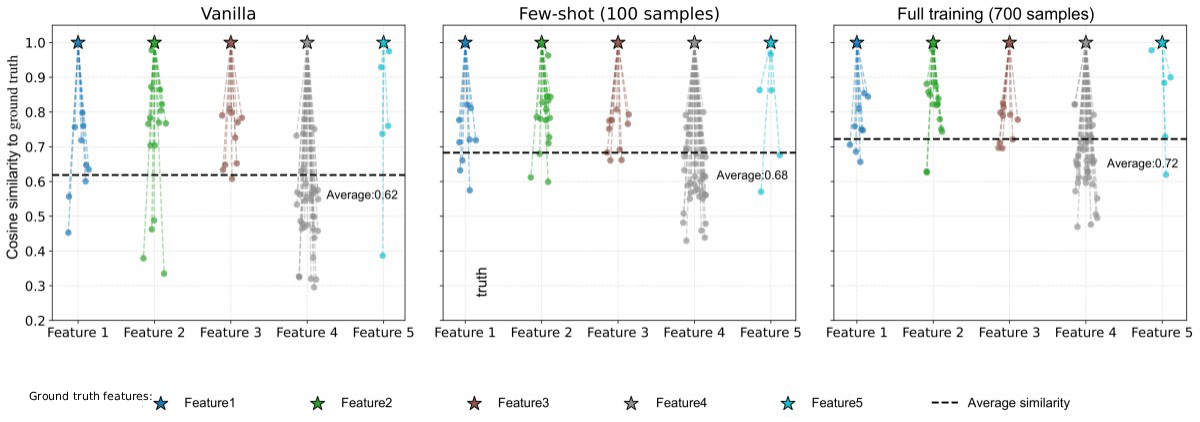
Cosine similarity between 5 representative features (stars) and their ground-truth features (dots).

We extend the analysis of the model’s capability to generalize through a complementary evaluation, which evaluates the embedding similarity between each feature and all its ground truth annotations. Each model has its own vocabulary vector that can be represented in high-dimensional space. Cosine similarity is used to measure how close each vocabulary vector is to the others semantically. As illustrated in Figure 3, we measured the cosine similarity distributions for 5 representative features across different model trainings to measure how the cosine similarity of the features to their ground truth changed. The vanilla model is the Mistral Nemo instruct model, where no training was conducted; only ICL with a few examples was used to guide model predictions. This approach achieved an average similarity of 0.62, indicating that modest inherent medical knowledge from the pretrained weights is brought into the model. The few-shot model improves that alignment up to 0.68, while the full model reaches 0.72, with tighter clustering around the ground-truth representations. Such progressive improvement demonstrates that semantic alignment is indeed vastly improved with fine-tuning, even with just 10 examples per case and with significantly less output variance.

The findings, therefore, underscore the promise of domain adaptation using our two-phase instructing and confidence-regularization fine-tuning setup, especially in low-resource clinical environments where obtaining labeled data may be a hard task. Having achieved these significant gains with merely 100 patient notes, which correspond to around 10 examples per clinical case type, only 100 records out of 700 (14.2% of the full dataset), further implies the viability of our approach in restricted medical domains where extensive, fully annotated datasets are often not available. To answer our first RQ1: “Is the model able to generalize effectively from limited training data, while maintaining competitive performance against full-data training?” the evidence strongly supports “yes.” Our system, with a few samples, having been trained using only 100 out of 700 patient notes (14.2% training set), obtained an *F*_1_-score of 0.973, which is about 99% of the full model performance (*F*_1_=0.983), and this is higher than previous state-of-the-art approaches working with fully annotated data. This showcases excellent generalization ability under minimal supervision, thereby affirming that our method can considerably reduce annotation demand while keeping high performance.

To measure whether the improvement observed by confidence-regularization fine-tuning over instructing fine-tuning is genuine, we used bootstrap resampling and McNemar test. Bootstrap resampling estimated the *F*_1_-score difference by repeatedly sampling the test set, yielding a 0.0235 difference with a 95% CI of 0.0175-0.0302 and a *P* value of *P*<.001, indicating a stable, significant improvement. McNemar test, analyzing paired outcomes, yielded a *P* value of .016, showing that the confidence-regularization model corrects more errors (124 features) than the instructing model (88 features). Both models were trained on 100 records and evaluated on 200 patient notes from the public dataset. These results confirm confidence-regularization model’s robust improvement over instructing fine-tuning model.

### Error Reduction Analysis (RQ2)

**Figure 4:**
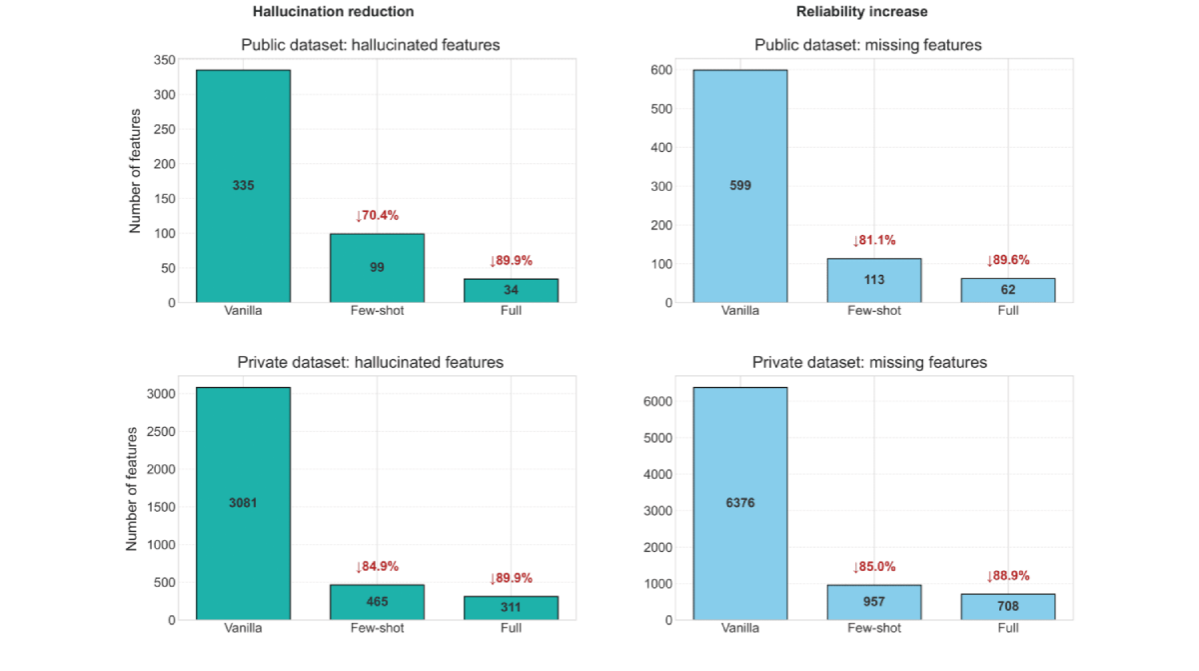
Comparison of missing and hallucinated features across model types. The vanilla model (Mistral Nemo with few-shot in-context learning [ICL]) shows the highest error rates, while the full model minimizes both error types.

To evaluate the capability of our confidence-regularization framework to reduce hallucination and improve reliability, we conducted two analyses: one measuring error distributions across model variants and the other measuring the evolution of hallucination and missing penalty functions during training epochs. All models—vanilla baseline, instructing fine-tuned, and confidence-regularization fine-tuned—were evaluated using the same inference pipeline (few-shot ICL + matching gate validation) to ensure fair comparison. Figure 4 quantifies the reduction in hallucination (left) and missing information (right), on both data splits, public (up) and private (down). The vanilla model (Mistral Nemo) incurs significant error rates, with 599 missing features (20.9% of 2861 features) and 335 hallucinated features (11.7% of 2861). The few-shot instructing fine-tuning model brings down these errors dramatically to 113 missing features (3.9% of 2861, a reduction of 81.1%) and 99 hallucinated features (3.5% of 2861, a reduction of 70.4%).

The full confidence-regularization model reduces these errors further to 62 missing features (2.2% of 2861, a reduction of 89.6%) and 34 hallucinated features (1.2% of 2861, a reduction of 89.9%).

In this respect, the pattern is consistently extended to the much larger private dataset (20,360 features), which misses a total of 6376 features (31.3% of 20,360) in addition to 3081 hallucinated features (15.1% of 20,360) using the vanilla model. The few-shot instructing fine-tuning model reduces the missing features to 957 (4.7% of 20,360, a reduction of 85%) and 465 hallucinated features (2.3% of 20,360, a reduction of 84.9%), while the full model misses only 708 features (3.5% of 20,360, a reduction of 88.9%) and 311 hallucinated features (1.5% of 20,360, a reduction of 89.9%).

**Figure 5:**
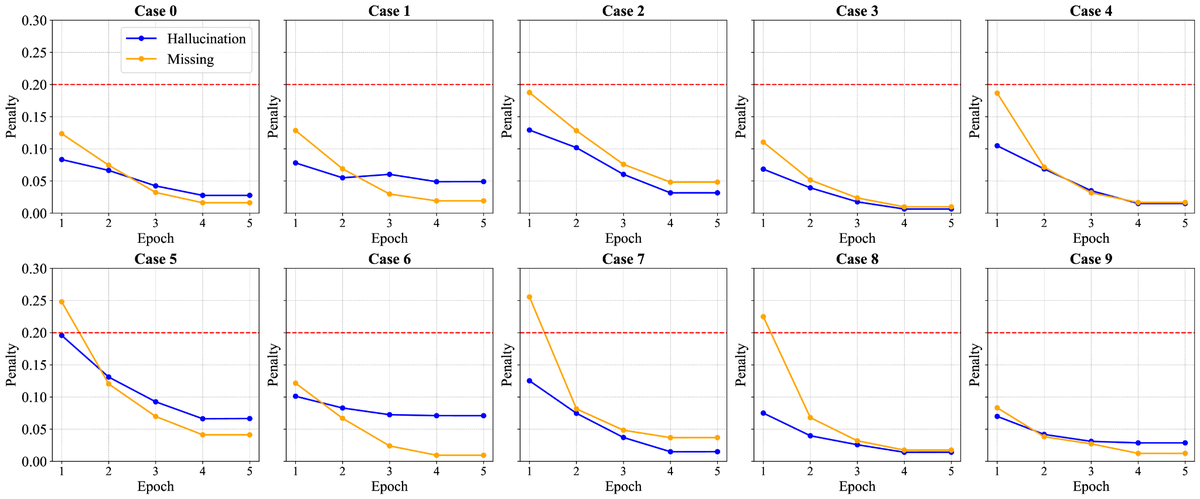
Evolution of hallucination (blue lines) and missing feature (yellow lines) penalty values over 5 training epochs for 10 clinical cases on the training dataset. The convergence of penalties illustrates the effectiveness of bidirectional semantic matching in balancing error types. The red line serves as a visual aid, highlighting where most penalties are <0.2.

To better understand the underlying mechanisms, Figure 5 presents the penalty trends across training epochs for all 10 clinical cases. The hallucination penalty (blue lines) consistently decreases as training progresses, with most cases showing a steep decline during epochs 1-3. Several interesting patterns emerge in the penalty curves: (1) missing feature penalties (yellow lines) often start higher than hallucination penalties, particularly in complex cases (cases 2, 5, 7, and 8), indicating an initial model bias toward precision over recall; (2) by epoch 5, nearly all cases demonstrate convergence to low penalty states (<0.05), showing the model has effectively handled feature detection and hallucination avoidance; and (3) the balanced convergence of both penalty types validates our bidirectional mapping approach, which prevents optimization from favoring one error type over another.

Being able to significantly reduce missing features and hallucinated features and, maintain similar reductions in penalty convergence across clinical cases proves beyond a doubt that our confidence-regularization framework robustly addresses RQ2. The proposed framework has been successful in minimizing hallucination problems prevalent to LLMs in medical contexts and in improving the reliability of extraction even in extremely low supervision (few-shot instructing fine-tuning).

### Calibration and Reliability (RQ3)

LLMs have proven to be powerful tools, with inherent deep contextual understanding capabilities, to process and generate human-like text. These models are often poorly calibrated due to their complex architecture and large number of parameters. Many previous works report that further instructing fine-tuning for specific task can improve accuracy but harm calibration, causing LLMs to become overconfident [55,56]. Our confidence-regularization framework reveals an unexpected calibration pattern. Rather than the typical confidence inflation seen in fine-tuned LLMs, our approach maintains stable average confidence (0.84, SD 0.003) while achieving dramatic performance improvements (*F*_1_ improved from 0.819 to 0.986). This creates a unique scenario where ECE increases not due to overconfidence, but from the growing misalignment between conservative confidence estimates and exceptional performance.

**Figure 6:**
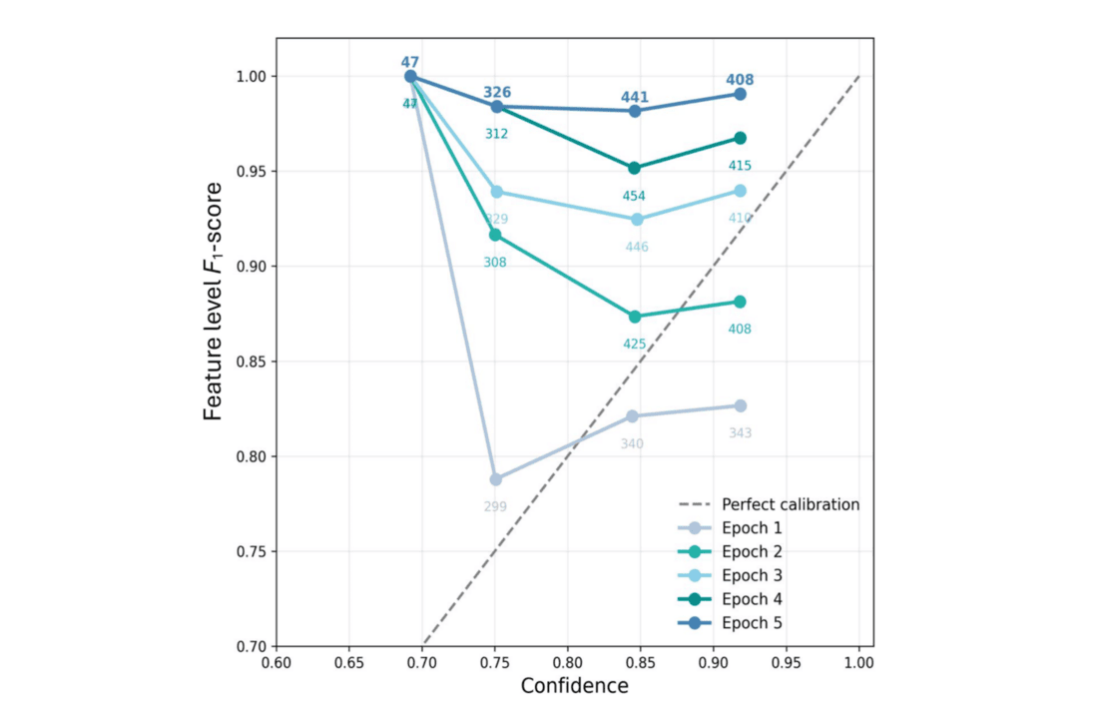
Reliability diagram showing feature-level performance as F1-score with softmax confidence per feature.

Figure 6 presents the reliability diagrams for our confidence-regularization model across 5 training epochs. Following the standard approach established by Guo et al [16], we partition predictions into 4 confidence bins (0.6-0.7, 0.7-0.8, 0.8-0.9, and 0.9-1.0) and compare the mean confidence within each bin against the actual *F*_1_ performance. Perfect calibration would result in all points lying on the diagonal line (y=x). As training progresses from epoch 1 to epoch 2, the model shows mixed calibration with significant underconfidence in the 0.6-0.7 bin; the gap between the average *F*_1_ score of this bin and optimal performance can be calculated as 0.692−1=−0.308 (negative mean underdominance). From epoch 3 to epoch 5 as *F*_1_ performance improves to 0.94-0.98, stable confidence becomes increasingly conservative. The 0.8-0.9 bin shows growing underconfidence (gap evolving from −0.077 to −0.136), while the highest confidence bin (0.9-1.0) transitions from slight overconfidence to underconfidence (gap evolving from 0.092 to −0.072). The distribution of predictions shifts toward higher confidence bins (from 683 to 849 predictions in the 0.8-1.0 range), but this reflects improved feature extraction quality rather than confidence inflation. In the highest confidence bin, *F*_1_-scores reach 0.98 by epoch 5, yet the model maintains conservative confidence estimates (SD 0.003). Despite the degradation in calibration, the model achieves remarkable performance improvements. *F*_1_-scores in the highest confidence bin (0.9-1.0) increase from 0.81 to 0.98, demonstrating that while calibration worsens, the model performance increases.

Our ECE increases from 0.060 to 0.147 across epochs, while the Brier score improves from 0.087 to 0.036. This is explained by our stable confidence mechanism: ECE measures the gap between confidence and performance, which naturally widens as performance improves while confidence remains constant. The improved Brier score indicates better probabilistic predictions despite the calibration mismatch. Contextualizing within medical NLP literature, Oliveira et al [57] reported ECE values for entity and relation extraction tasks ranging from 0.12 to 0.44, depending on the dataset and strategy. Our final ECE of 0.147 falls within this expected range. The increasing ECE aligns with findings from another study [20], which show that pretrained language models do not learn to become calibrated during training; they become increasingly overconfident. In our case, penalties lead to conservatism rather than overconfidence.

This “stable confidence with improving performance” pattern addresses (RQ3) in an unexpected way. The confidence-regularization strategy has not yet achieved tight calibration; instead, it prevents the overconfidence seen in fine-tuned LLMs. Following the framework of Xiao et al [58], our model operates in what they term the “non-calibratable regime,” where performance increases as ECE increases. However, unlike typical models in this regime that exhibit severe overconfidence, our approach maintains conservative, stable confidence estimates.

For medical applications, this represents a desirable trade-off. Conservative confidence estimates (underconfidence) are preferable to overconfidence when errors have serious consequences.

Given the reliability diagram and rising ECE, our findings corroborate a growing body of published evidence that alignment improves task quality at the expense of calibration. It enters the “non-calibratable regime” described by Xiao et al [58], where further accuracy gains cannot be matched by improvements in calibration. Our inference-time gating offers a viable, low-cost alternative when the task requires matching with existing references, as the case in feature extraction for this specific task.

### Qualitative Error Analysis

**Figure 7:**
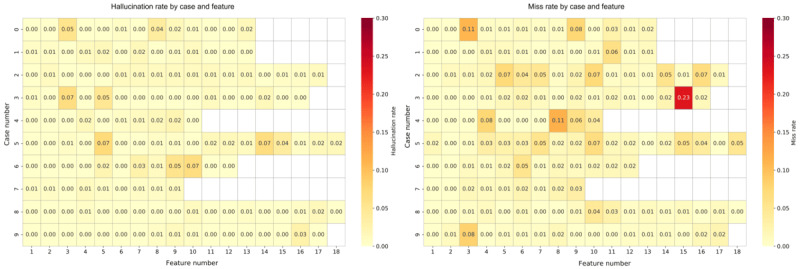
Heatmap showing hallucination and omission (missing features) rates for all clinical features in the private test set, where darker red indicates higher error rates and light yellow indicates lower error rates.

We analyzed our model’s predictions on a private dataset containing 26,297 features. The hallucination rate is defined as precision (FP/[TP+FP]) and the miss rate as recall (FN/[TP+FN]) per feature. The heatmap in Figure 7 shows these features, with darker colors indicating higher hallucination (precision) and missing feature (recall) error rates. Our analysis shows that hallucination errors occur less frequently than missing feature errors, with the highest hallucination rates at 7%, while missing features showed an even higher occurrence, up to 23%.

Upon closer examination of these features (detailed examples provided in Multimedia Appendix 2), we identified several consistent patterns, as illustrated below.

#### Hallucination Error Patterns

##### Overview

Annotation inconsistencies: many features categorized as hallucinations stem from inaccuracies in the ground truth annotations. For example, the feature “worsened 3 weeks ago” was not in the ground truth of one patient note, while an equivalent feature, “symptoms worsened 3 weeks,” was annotated as ground truth in another note belonging to the same case number.

##### Specificity requirements

Certain features demand exact descriptions of symptoms to be identified accurately. For example, the feature “Epigastric-discomfort,” where epigastric specifically refers to the upper central region of the abdomen, was annotated in ground truth as “pain in the middle of his stomach.” On the other hand, the model’s prediction of a nonspecific “stomach pain,” lacking details on the precise location, caused the predictions to fail similarity matching and be registered as hallucinations.

##### Redundant documentation

In several cases, features were listed twice in patient documentation using varied phrasing but were annotated only once. For example, the predicted feature “Denies dyspnea” was present in the text but not considered during inference because annotators had only marked “negative shortness of breath.”

##### Knowledge limitations

A significant error example represents a fundamental knowledge gap inherent in the LLM, such as “No-relief-with-asthma-inhaler,” predicted as “tylenol did not help.” Tylenol is a pain reliever, not an asthma medication.

#### Missing Feature Error Patterns

Predicting complex features proved consistently challenging for the model. As shown in Tables S3 and S4 in Multimedia Appendix 2, for compound features like “No-hair-changes-OR-no-nail-changes-OR-no-temperature-intolerance,” the model predicted only “denies change in weight,” while the actual annotation for this patient was “Denies any sweating.” Similarly challenging was the feature “Lack-of-other-thyroid-symptoms,” which lacks standardization and was inconsistently captured by annotators.

Our analysis identified 4 key limitations: annotation constraints, where human annotators showed inconsistencies in the annotated data, as evidenced by the agreement percentage [36], affecting model evaluation; semantic depth disparity, where the model’s deep semantic understanding was sometimes interpreted differently from the annotation team’s standardized approach, leading to instances of false positive results; feature ambiguity, where some features were rather generic in nature and lacked a clear set of well-defined boundaries, making consistent annotation and prediction difficult; and model internal knowledge gaps, where the model’s knowledge limitations caused errors such as confusing medication classes (eg, pain relievers vs bronchodilators) or anatomic localizations (eg, epigastric vs general stomach pain). The error analysis underscores that the annotation process lacks comprehensiveness and standardization, in addition to being subject to human error bias and the complexity of annotating large text volumes, making it a challenging task. LLMs, highly sensitive to data quality, require consistent, standardized training data for optimal performance, and the model’s medical domain knowledge limitations could be improved with accurately annotated data and continuous training on domain-specific medical data to significantly enhance performance.

## Discussion

### Overview

Our framework efficiently extracts medical features from patient notes by integrating diverse training approaches, multidimensional confidence-regularization, and embedding similarity evaluation. It achieves a state-of-the-art *F*_1_-score of 0.983 (semantic matching) and 0.968 (binary overlap) on the full dataset, and 0.973 (semantic matching) and 0.952 (binary overlap) with only 100 patient notes, outperforming benchmark models INCITE (*F*_1_=0.888) and DeBERTa-based models (*F*_1_=0.958). The framework provides a transformative solution for automated medical education assessment, addressing data scarcity, semantic variability, hallucination risks, and interoperability.

The framework’s strength lies in its strong generalization, retaining 99% performance with only 100 out of 700 patient notes (14.2% of the training data) through instructing and confidence-regularization fine-tuning. Semantic embedding matching captures variable clinical expressions (eg, “worsen 3 wks ago” vs “symptoms worsened 3 weeks”), unlike token-level probability methods. Our framework ensures performance stability, reducing overfitting in data-scarce settings—crucial for resource-constrained environments where manual annotation is costly.

Unlike traditional n-gram, fuzzy logic, or BERT-based methods, which struggle with semantic variability in clinical notes, our framework adapts semantic embedding matching and lightweight sentence embedding for cosine similarity, capturing deeper semantic meaning.

Our confidence-regularization fine-tuning phase significantly enhances feature extraction performance, reducing hallucinations by 89.9% (from 3081 to 311) and missing features by 88.9% (from 6376 to 708). However, this phase does not improve the ECE, which increases from 0.060 to 0.147 over 5 training epochs. This trade-off arises not from overconfidence but from the growing misalignment between conservative confidence estimates and exceptional performance. Despite the well-documented overconfidence and the miscalibration of LLMs [55,56], our approach maintains stable, conservative confidence, which becomes underconfident as performance improves.

With near-human performance, the framework evaluates 200 notes in 30 minutes on a high-performance GPU, reducing manual effort for USMLE Step-2 Clinical Skills examination preparation. Its few-shot learning and modular architecture eliminate the need for span annotation (interrater agreement of 84%) and enable customization. The framework shows promise for extracting structured data from electronic health records, diagnostic reports, and discharge notes, improving decision-making in health care.

Compared to lexicon-based INCITE, BERT-based models, or DeBERTa with pseudo-labeling, our framework achieves higher precision and recall through two-phase fine-tuning, with both semantic matching (0.988 vs 0.961 precision; 0.978 vs 0.963 recall) and binary overlap evaluation. Our inference-time matching gate effectively replaces traditional calibration needs through context-aware filtering. Unlike post hoc calibration requiring held-out data (which would reduce our already limited training set), our approach validates extractions against case-specific feature lists. This addresses a unique challenge in medical feature extraction: identical text (eg, “fever”) may be correct for case 1 but incorrect for case 2, making global calibration inappropriate.

Our framework generates output that supports human-in-the-loop deployment in medical education assessment. During inference, for each case-specific feature that needs to be identified, the system produces: (1) a matching gate decision indicating whether the feature was found in the patient note (present/absent), (2) the extracted text segment and its offset, and (3) a cosine similarity score (0-1 range) between the extracted text and ground truth during testing, which can also be applied to the case’s target feature during deployment. These outputs enable medical instructors to review the output by identifying weakly matched extracted features with low cosine similarity scores that may need manual rechecking. Missing features for features from the case list that the model could not find (marked absent) are highlighted for instructor review to confirm they are truly missing. Potential hallucination occurs when the model predicts a feature that could not be found by the matching gate, indicating incorrect extraction. For deployment, institutions can customize review thresholds based on their requirements. High-stakes examinations might mandate manual review of all extractions with cosine similarity >0.8 to their target features, while formative assessments could limit review to missing features only. This flexibility ensures the system augments rather than replaces human judgment in educational assessment.

### Alignment With Artificial Intelligence Trustworthiness Framework

CONSORT-AI (Consolidated Standards of Reporting Trials–Artificial Intelligence) guidelines provide a complete checklist for any artificial intelligence (AI)–based clinical intervention, ensuring transparent and reproducible reporting of AI usage during clinical practice [59]. In the following subsections, we detail how the proposed framework meets important key requirements of CONSORT-AI.

#### Clear Description of AI Intervention (Item 5)

CONSORT-AI requires a detailed description of the AI intervention, such as how it is used and integrated into clinical workflows. Our framework is used for automating the assessment process of medical feature extraction from clinical examination notes written by medical students. The framework’s two-phase approach is clearly described, with detailed algorithms (Algorithm 1) and hyperparameters in Multimedia Appendix 2 to ensure reproducibility. Its intended use is to support automated assessment in medical education, reducing the burden on human evaluators while maintaining high accuracy (*F*_1_=0.983). This framework is considered as part of the examination, where the examiner must also evaluate other communication skills.

#### Input and Output Specification (Item 7)

CONSORT-AI requires clarification of the input data and output format of the AI system. Input data are described in (Phase 1: Instructing Fine-Tuning section), detailed data collection and annotation prepared by NBME [36]. To prepare data for training the model, notes were descoped and formatted using standardized instructions and few-shot ICL (4-6 exemplar pairs, as described in the section Phase 1: Instructing Fine-Tuning). The output consists of patient notes, features associated with patient note cases, extracted features validated through the matching gate, similarity score, and the matched part within patient notes, ensuring outputs are structured and clinically relevant.

#### Reproducibility and Code Availability (Items 10-11)

Based on CONSORT-AI, details ensuring reproducibility and providing access to code and data are required. The complete source code, including training and inference code, is available in a GitHub repository [62]. Model weights and training protocols are fully documented. The dataset is accessible through Kaggle and NBME data-sharing agreement. Computational requirements are specified (NVIDIA H100 GPUs).

#### Performance and Error Reporting (Item 12)

CONSORT-AI encourages comprehensive reporting of performance metrics and error types. Our proposed model provides detailed performance metrics (precision, recall, and *F*_1_-score) across public and private dataset splits (Table 2), achieving *F*_1_=0.983 with full data and *F*_1_=0.973 with 100 notes. Error analysis investigating the pattern and reasons of hallucination and missing features is provided in the Qualitative Error Analysis section.

#### Handling of Errors and Failures (Item 19)

CONSORT-AI requires describing how errors and failures are managed. During training, errors are detected using bidirectional semantic matching (in step 3 of the Phase 2: Confidence Regularization Fine-Tuning section), targeting hallucinations and missing features. The inference-time matching gate ensures filtering of nonexistent predictions, supporting reliable clinical outputs. During deployment, the model extracts features and displays the relevant feature numbers, allowing human intervention by tracking the extraction process.

#### Human-AI Interaction (Item 20)

CONSORT-AI stresses the role of human oversight in AI systems. Our framework supports human oversight through a cosine similarity score for each extracted feature, along with the number and text of the feature associated with the case, enabling experts to prioritize review of features below a set low cosine similarity threshold. The matching gate facilitates verification by eliminating mismatched features. All extracted features are matched with patient note text then approved to be passed. This creates oversight transparency where the errors can be tracked in text. Integration with electronic health record systems could further allow clinicians to accept, reject, or flag model outputs prior to downstream use. At present, this capacity is not implemented in the prototype but is identified as a key future development for clinical deployment.

#### Validation in Clinical Context (Item 22)

CONSORT-AI requires validation of AI performance in intended clinical contexts. The proposed framework’s evaluation on the USMLE Step-2 dataset (2839 notes) demonstrates applicability in medical education assessment. Its ability to maintain high performance with minimal data (100 notes, 14.2% of training data) supports scalability in resource-constrained settings.

By following CONSORT-AI guidelines, the proposed framework ensures transparency, reliability, and applicability, making it a trusted option for automated medical feature extraction in clinical education environments.

### Limitations

While our framework demonstrated strong performance, the evaluation shows several shortcomings that highlight potential areas for improvement.

First, inconsistencies in human-annotated ground truths, such as ambiguous boundaries, subjective symptom consideration, and missing annotations, led to hallucination and missing feature errors. Developing standardized annotation methodologies and clear clinical feature definitions is essential to enhance annotator agreement.

Second, the LLM backbone demonstrates general medical knowledge by performing well with only few-shot fine-tuning but struggles with specific medical logic, such as medication-symptom relationships. Continual pretraining on medical domain data, integrating structured medical ontologies (eg, Systematized Nomenclature of Medicine–Clinical Terms, Unified Medical Language System), or using retrieval-augmented generation could address these gaps.

Third, designing model training on medical datasets may further improve extraction performance. Testing on USMLE Step-2 Clinical Skills notes provided a controlled case collection, but the framework lacks evaluation on unstructured or multilingual health care records, such as electronic health records or notes from psychiatry and pediatrics. Future work could consider semantic adjustments for multilingual records to enhance global applicability by incorporating continual pretraining or multitask fine-tuning.

Fourth, despite the competitive performance of our framework, it demonstrates the typical calibration-accuracy trade-off observed in fine-tuned LLMs [20,55,56,58]. ECE increases from 0.060 to 0.147 during training. Although this pattern aligns with existing literature showing that task-specific fine-tuning often harms calibration [61], our model shows stable confidence, which contrasts with literature observations that LLMs tend to be overconfident. The model’s inability to adjust confidence upward as performance improves from 0.82 to 0.98 suggests that our regularization may be overly restrictive, representing a limitation for applications requiring well-calibrated confidence scores.

Fifth, while our framework and baseline models [9] both use binary feature-detection *F*_1_ metrics for evaluation, the method of determining feature presence differs between approaches. The baseline models use token-level probability thresholds (≥0.5) from span-extraction architectures, which is inapplicable to generative models that produce textual descriptions. We therefore implemented two adapted evaluations: (1) binary overlap using token overlap ≥0.5 between generated and ground truth text, providing the closest equivalent to baseline methodology, and (2) semantic matching using cosine similarity ≥0.5 to capture semantic equivalence. The semantic approach is inherently more lenient than token-based methods, which may introduce a positive bias. However, our binary overlap results (*F*_1_=0.968) still demonstrate improvements over baselines (*F*_1_=0.958), confirming performance gains independent of evaluation methodology.

Sixth, our framework is specifically designed for structured extraction tasks with predetermined feature lists, as demonstrated in the USMLE Step-2 Clinical Skills evaluation. The framework’s evaluation pipeline requires both matching gate validation and cosine similarity comparison (≥0.5) against target feature descriptions to determine feature presence. Without predefined target features, the cosine similarity component cannot function, invalidating our current evaluation methodology. While the core confidence-regularization training objective could potentially be adapted to open-ended named entity recognition tasks through span-level token overlap *F*_1_ scoring, this would require a fundamental redesign of the validation pipeline and evaluation framework. Such adaptation represents a distinct research direction requiring substantial methodological changes rather than a straightforward extension of the current work.

Seventh, future work should systematically investigate the sensitivity of confidence-regularization parameters across diverse medical domains and backbone architectures. While our configuration (α=0.7, β=0.2, λ=20, τ=0.6-0.7) achieved stable convergence on the USMLE dataset, understanding the parameter sensitivity ranges would provide practical guidelines for adapting this framework to new clinical NLP tasks with different precision–recall characteristics and error distributions.

Eighth, we demonstrate our two-phase framework on the Mistral-NeMo-Instruct-2407 backbone. Our choice of this backbone was task-driven, based on preliminary experiments on this dataset [37]. While this open model supports reproducibility, we did not perform systematic cross-backbone evaluation (eg, public Mistral-7B-Instruct, Llama-3-Instruct). Thus, the extent to which our gains translate to other model families remains to be quantified and is a direction for future work.

### Future Directions

Building on the proposed framework’s foundation, future research will focus on advancing its architectural capabilities to enhance the precise identification of clinical features in text, a limitation that has sparked significant interest across the LLM community. While traditional autoregressive models are effective for semantic generation, they struggle with determining exact token positions, reducing their effectiveness in span-sensitive tasks such as named entity recognition and clinical information extraction. This has motivated a wave of research investigating alternative training approaches that can use bidirectional generation, encoder-decoder, and matching-based retrieval models.

We plan to investigate accurate token-level positional grounding while exploring architectures focused on matching and training objectives that can enhance token-level grounding between input and output. Domain-specific pretraining on clinical corpora such as Medical Information Mart for Intensive Care III [60] can further support the model’s understanding of medical terminologies.

We will further evaluate our framework across a wider range of clinical settings, such as unstructured electronic health records, radiology reports, and multilingual patient records. Using standardized medical ontologies, such as Unified Medical Language System and Systematized Nomenclature of Medicine–Clinical Terms, can enhance annotation uniformity and boost span-level accuracy. An in-depth study of poor calibration in LLMs is warranted, which may involve architectural adjustments to address persistent overconfidence. Such changes could entail revising attention mechanisms or implementing dedicated calibration layers to more effectively model uncertainty estimates for feature extraction tasks that depend on contextual features that change for each case.

### Conclusions

This study developed and evaluated a two-phase LLM framework for automated medical feature extraction from clinical examination notes. Our approach consisted of two training phases: instructing fine-tuning, followed by confidence-regularization fine-tuning, to address the critical challenges in medical NLP applications, including data scarcity, semantic variability, and hallucination risks.

The framework achieved an *F*_1_-score of 0.983 (semantic matching) and 0.968 (binary overlap) on the USMLE Step-2 Clinical Skills dataset with full training data and maintained an *F*_1_-score of 0.973 (semantic matching) and 0.952 (binary overlap) using only 100 out of 700 training samples (14.2% of the full dataset). Through the two-phase training approach, hallucinated outputs were reduced by 89.9% (from 3081 to 311) and missing features decreased by 88.9% (from 6376 to 708) compared to the baseline LLM model with few-shot ICL. While the confidence-regularization fine-tuning improved extraction accuracy, it increased the ECE from 0.060 to 0.147, reflecting the documented calibration–accuracy trade-off in fine-tuned LLMs. Our inference pipeline’s semantic similarity validation provides practical calibration by filtering predictions based on feature matching rather than raw confidence scores.

This framework provides a practical solution for automated medical education assessment, reducing the burden on human evaluators while maintaining quality standards for clinical competency evaluation. The demonstrated ability to achieve strong performance with minimal training data positions this approach as a valuable tool for scaling medical education assessment in resource-constrained environments.

## Appendix

Multimedia Appendix 1 Confidence-regularization fine-tuning detailed formulas.

Multimedia Appendix 2 Algorithm 1, training specifications and error analysis samples.

## Abbreviations

AI: artificial intelligence
BERT: bidirectional encoder representations from transformers
chrF: character n-gram F1-score
CONSORT-AI: Consolidated Standards of Reporting Trials–Artificial Intelligence
DeBERTa: decoding-enhanced bidirectional encoder representations from transformers with disentangled attention
ECE: expected calibration error
GPU: graphics processing unit
ICL: in-context learning
INCITE: intelligent clinical text evaluator
LLM: large language model
NBME: National Board of Medical Examiners
NLP: natural language processing
RQ: research question
USMLE: United States Medical Licensing Examination

## Data Availability

The data sets analyzed during the current study are available in the Kaggle competition [37]. The complete data sets, including both public and private splits, are not publicly available due to licensing restrictions but are available from the National Board of Medical Examiners through a data-sharing agreement [63]. The source code generated during this study is available in a GitHub repository [62].
