# Supplementary material for: Medical Feature Extraction From Clinical Examination Notes: Development and Evaluation of a Two-Phase Large Language Model Framework
Source: JMIR Med Inform. 2025 Dec 3;13:e78432. doi: 10.2196/78432 (PMC12712565; doi:10.2196/78432)
Supplement: Multimedia Appendix 1 [file medinform_v13i1e78432_app1.doc]

These two sections: Confidence Estimation, and confidence adaptive training illustrate detailed description of Confidence-regularization fine-tuning phase.

### **Confidence estimation**

The first step in confidence calibration involves obtaining the confidence of generated features. As the model is autoregressive, confidence is estimated using token-level probabilities from the softmax layer. For each extracted feature
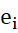
, we calculate an initial confidence score
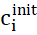
 using the sequence of generated tokens
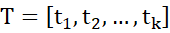
 and their associated token-level probabilities
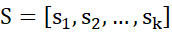
.

The token probabilities are averaged over the extracted feature's duration to approximate the initial confidence score:


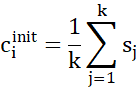


We normalize the confidence based on the length of the feature because the joint likelihood of longer sequences tends to produce lower confidence distributions; to address the diverse lengths and semantic depth of extractions, we implement a complexity adjustment factor, expressed as:


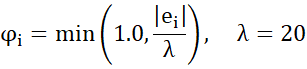


where
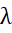
 is a scaling parameter set to 20, which represents the character length threshold beyond which features are considered maximally complex, and
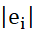
 denotes the character length of the extracted feature.

The initial estimate and the complexity factor are combined to calculate the complexity adjusted confidence score:


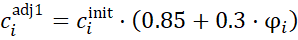


This formula inspired by proper scoring rules used in calibration theory, such as the Brier score and negative log-likelihood , but reinterpreted through a soft penalty lens. Specifically:

- The baseline multiplier 0.85: penalizes overconfident predictions on short or trivial features, consistent with findings that neural models tend to be overconfident in high-frequency outputs.
- The complexity term
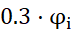
: acts as an input-dependent uncertainty correction, reflecting aleatoric variability as discussed in Bayesian deep learning.

The scaling factor range of 0.85 + 0.3 * φᵢ (φᵢ ∈ [0, 1]) balances conservatism and specificity. It reduces confidence by 15% for simple features (e.g., "fever," scaling = 0.925) to avoid overconfidence and boosts it by up to 15% for complex, specific phrases (e.g., "persistent bilateral lower extremity edema," scaling = 1.15). This ±15% range, starting from a conservative 0.85 baseline, ensures cautious yet meaningful adjustments, maintaining calibration while prioritizing specificity in medical applications.

### **Confidence adaptive training**

#### **Confidence Feedback Loop**

To stabilize confidence estimates across training, we incorporate a feedback mechanism that blends current token-derived confidence scores with the model’s historical performance. At each epoch
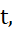
 we maintain a running average
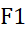
 score, represented as
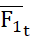
, calculated over all training examples. This metric guides the adjustment of the complexity-adjusted confidence score
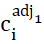
 for each feature extracted calculated using:


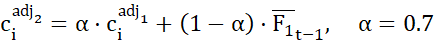


where
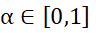
that controls the trade-off between the model's current confidence and its historical performance; in our implementation, this coefficient is set to 0.7. to favor current uncertainty estimates while still incorporating empirical stability from prior epochs. This formulation draws inspiration from Bayesian updating and momentum-based optimization, where past statistics are used to stabilize noisy point estimates.

### **Controlled Stochasticity**

To prevent the collapse of confidence scores into narrow ranges, we introduce controlled stochastic randomness to feature complexity. to generate the final confidence score:


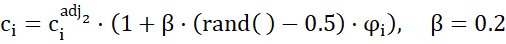


Here, rand() is a uniformly distributed random variable between 0 and 1, and the term


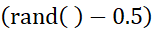
ensures the noise is zero-centered, producing symmetric variation in the range ±0.5%. No fixed seed is applied, as the randomness is deliberately injected at runtime to simulate aleatoric uncertainty. With
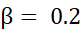
 offering controlled variability centered around, adjusted confidence, the parameter
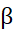
 regulates the amount of injected randomness to ±10% for maximally complex features where
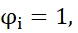
while shorter features receive proportionally less noise. This design is inspired by Bayesian learning uncertainty modelling techniques like Monte Carlo dropout , where underlying ambiguity in generation tasks is captured by mild stochasticity.

### **Progressive Threshold Adjustment (Calibration Curriculum)**

To align confidence penalties with the model's maturity during training, we adopt a progressively increasing confidence threshold. This approach follows a curriculum learning paradigm, where early stages of training tolerate overconfidence, while later stages enforce stricter calibration. Formally, the threshold at epoch 𝑡 is computed as:


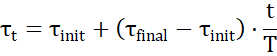


where
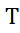
 is the total number of training epochs,
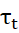
 is the confidence threshold at epoch
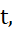
 where
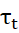
 is the confidence threshold at epoch
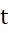
,
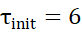
 and
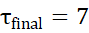
 are the initial and final threshold values, as training goes on. This approach allows the model to gradually adjust to more stricter calibration requirements throughout the training process.

**Training Objectives**

Our training methodology employs multiple objectives to balance model extraction ability, reliability, and completeness.

1. **Base Language Modeling Loss**

The foundation of our training approach is the standard language modeling loss:


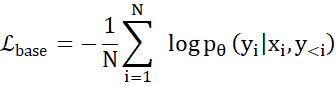


where
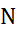
 is the total number of tokens in the output sequence, and
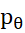
 is the probability that the model with parameters
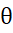
 will assign to the correct token
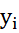
 given the input
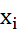
 and prior tokens
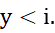


1. **Hallucination Penalty**

As a regularization approach applied during training model is applying penalty on overconfidence wrong prediction. Following proper scoring rule theory by use of quadratic error terms mirrors the Brier score, which penalizes the square of the difference between predicted probability and actual outcome. We introduce an F1-based confidence penalty that penalizes incorrect extractions proportionally to their confidence and based on F1 as performance matrices:


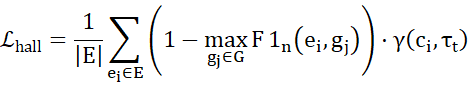


where
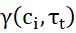
 is a confidence scaling function defined as:


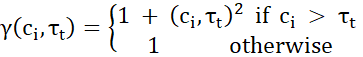


This design imposes a quadratic penalty on overconfident errors, thereby promoting reliability in line with the Brier score's reliability term and proper scoring rule principles. The progressive threshold
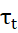
 makes this penalty adaptively stricter as the model matures, forming a curriculum-like pressure toward calibration.

1. **Missing Feature Penalty**

To address missing features, we introduce a complementary penalty based on the backward mapping mechanism:


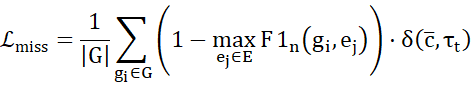


where
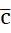
 is the average confidence across all extractions, calculated as:


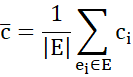


and
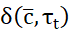
 is defined as:


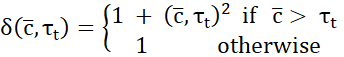


This penalty increases when the model is generally overconfident but misses important features, addressing the dual problems of hallucination and missing features.

1. **Combined Training Objective**

Our final training objective combines these components with appropriate weighting:


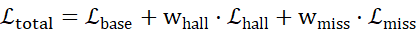


where the hyperparameters
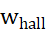
and
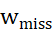
 regulate the respective contributions of the hallucination and missing feature penalties. We set
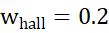
 and
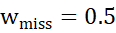
 based on validation performance in our implementation where we need to increase recall. Each penalty term is only applied when the model’s confidence for an incorrect extraction exceeds the current threshold value; otherwise, no penalty is computed for that case. This integrated objective creates a well-balanced optimization environment that enhances feature extraction accuracy, mitigating hallucination. and missing feature for effective and accurate feature extraction.
