# Supplementary material for: Medical Feature Extraction From Clinical Examination Notes: Development and Evaluation of a Two-Phase Large Language Model Framework
Source: JMIR Med Inform. 2025 Dec 3;13:e78432. doi: 10.2196/78432 (PMC12712565; doi:10.2196/78432)
Supplement: Multimedia Appendix 2 [file medinform_v13i1e78432_app2.doc]

# Appendix 1

## Confidence-regularization fine-tuning algorithm

This algorithm illustrates the steps of confidence-regularization fine-tuning training.

| **Algorithm 1** Confidence-regularization fine-tuning for medical feature extraction | |
| --- | --- |
| **Require:** Patient note 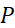, ground truth features 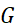, fine-tuned model 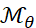, number of training epochs 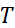, and Initial confidence threshold 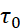 | |
| 1: | Initialize model parameters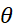, optimizer, and threshold schedule 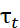 |
| 2: | **for** epoch index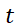 = 1 to 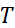 |
| 3: | **for** each training instance 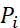 with gold features 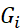 |
| 4: | Generate extracted features 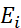🡨 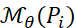 |
| 5: | Estimate token level confidence scores 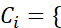 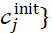from softmax probabilities over token in 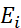 |
| 6: | Retrieve historical epoch-level F1 score: 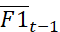 |
| 7: | **for** each extracted feature 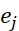 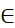 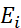 do: |
| 8: | Compute feature length complexity score 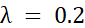: |
| 9: | 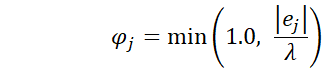 |
| 10: | Adjust confidence: |
| 11: | 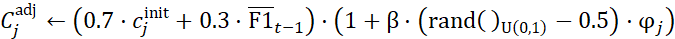 |
| 12: | **end for** |
| 13: | Perform semantic matching between 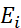 and 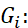 |
| 14: | - Forward: Compare each 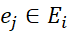 to all 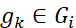 to detect hallucinated feature |
| 15: | - Backward: Compare each 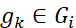 to all 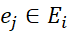 to detect missing feature |
| 16: | Loss Components: |
| 17: | - Compute hallucination penalty: |
| 18: | 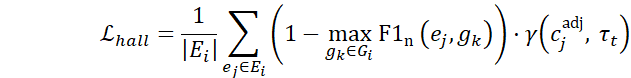 |
| 19: | - Compute missing feature penalty: |
| 20: | 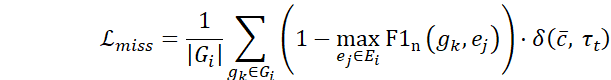 |
| 21: | Where: 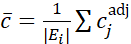 |
| 22: | - Compute base loss 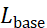 |
| 23: | Compute total loss: |
| 24: | 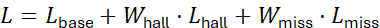 |
| 25: | Backpropagate 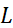 and update model parameters |
| 26: | **end for** |
| 27: | **return** Final fine-tuned model 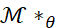 |

## Training Specification

For instruct fine tuning we integrate the instructions in Figure . In this phase we train two models few shot and full data, the training setting for both models appear in Table and Table .


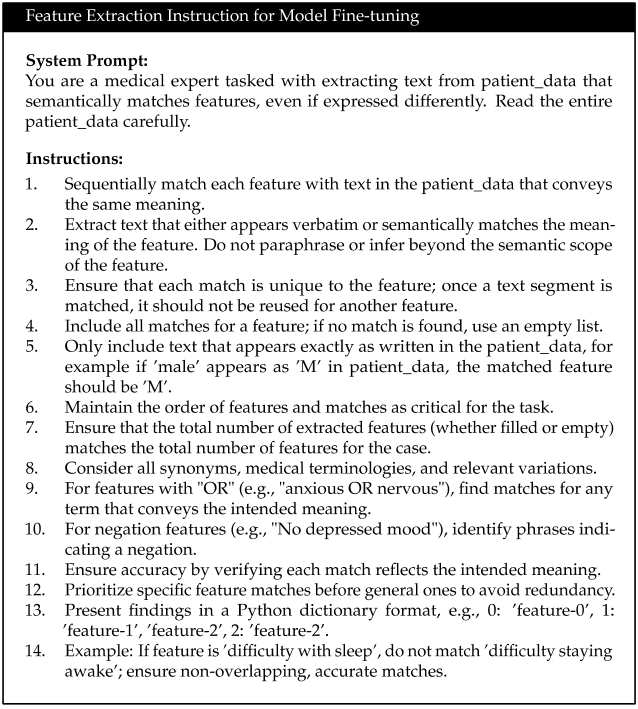


**Figure S1:** Detailed instruction template used for fine-tuning the model for medical feature extraction from patient notes[40].

## Error Analysis

These tables Table , Table This appendix presents detailed examples of model predictions that were categorized as either hallucinated or missing features during evaluation, specifically focusing on features with the highest error rates. Table illustrates three representative cases of hallucination errors. In each example, the left column contains a predicted feature that was either not present in the patient note or not annotated in the ground truth. The right column shows the ground-truth annotation for the same feature number in a different patient note from the same case, highlighting annotation inconsistencies or semantic mismatches.

**Table S1:** Training hyperparameters and configuration settings for instruct fine-tuning on few-shot training and full data training.

| **Parameter** | **Few-Shot Training** | **Full Data Training** |
| --- | --- | --- |
| **Model Configuration** | | |
| Base model | Mistral-Nemo-Instruct-2407 | Mistral-Nemo-Instruct-2407 |
| Max sequence length | 2048 tokens | 2048 tokens |
| Quantization | 4-bit quantization | 4-bit quantization |
| **LoRA Configuration** | | |
| LoRA rank (r) | 32 | 32 |
| LoRA alpha | 32 | 32 |
| LoRA dropout | 0.2 | 0.2 |
| **Training Configuration** | | |
| Training epochs | 14 | 7 |
| Learning rate | 2e-4 | 1e-4 |
| Optimizer | AdamW (8-bit) | AdamW (8-bit) |
| Weight decay | 0.01 | 0.01 |
| LR scheduler | Linear | Linear |
| **Hardware Specifications** | | |
| GPU | NVIDIA A100 | NVIDIA A100 |
| Training time | 0.5 hours | 1 hours |

**Table S2:** Training parameters for F1-based calibration tuning on few-shot training and full data.

| **Parameter** | **Few-Shot Training** | **Full Data Training** |
| --- | --- | --- |
| **Model Configuration** | | |
| Base model | Manal0809/Mistral_instructive_few | Manal0809/Mistral_instructive_full |
| Max sequence length | 11000 tokens | 11000 tokens |
| Quantization | 4-bit quantization | 4-bit quantization |
| **LoRA Configuration** | | |
| LoRA rank (r) | 32 | 32 |
| LoRA alpha | 32 | 32 |
| LoRA dropout | 0.2 | 0.2 |
| **Training Configuration** | | |
| Training epochs | 5 | 5 |
| Learning rate | 1e-4 | 1e-4 |
| Optimizer | AdamW (8-bit) | AdamW (8-bit) |
| Weight decay | 0.01 | 0.01 |
| LR scheduler | Linear with warmup | Linear with warmup |
| Warmup | 10% of steps | 10% of steps |
| **Calibration Parameters** | | |
| Hallucination weight | 0.2 | 0.2 |
| Missing feature weight | 0.5 | 0.5 |
| Confidence threshold | 0.7 | 0.7 |
| **Hardware Specifications** | | |
| GPU | NVIDIA H100 | NVIDIA H100 |
| Training time | 2.5 hours | 12 hours |

Table presents two representative features associated with high missing rates. These cases reflect inherent challenges in standardizing complex or compound clinical concepts. For instance, thyroid-related features often lack consistent phrasing across notes, leading to semantic ambiguity and lower extraction performance. Together, these examples underscore the importance of improved annotation consistency, clearer feature definitions, and architectural strategies to better capture clinical variability.

**Table S3:** Examples of hallucinated features with corresponding annotations from other notes in the same case and feature index.

| **Case 3: Epigastric-discomfort (Feature Number: 3)** | |
| --- | --- |
| Predicted Feature | Sample of Annotated Features |
| ['pain present for 2 mos', 'stomach pain'] | ['stomach pain that is his middle of chest'] |
| [‘pain is located in the stomach’] | ['pain in his upper mid epigastric area'] |
| ['stomach pain'] | ['pain in the epigastrum'] |
| ['pain in his upper abdomen'] | ['pain in the middle of his stomach'] |
| ['pain the middle of his upper abdomen'] | ['pain in upper abdomen'] |
|  |  |
| **Case 5: Increased-frequency-recently (Feature Number: 5)** | |
| Predicted Feature | Sample of Annotated Features |
| ['now it has begin for the past 3 wks'] | {['recently occurred more frequently in the past 3 weeks'] |
| ['getting worse'] |
| ['x 2 wk', 'one episode every few days'] | ['sypmtoms worsened 3 weeks'] |
| ['worsen 3 wks ago'] |
| ['multiple times throughout the day'] | ['past three weeks she has been getting one every 2-3 days'] |
| ['absent until 3 weeks ago'] |
|  | |
| **Case 6: No-relief-with-asthma-inhaler (Feature Number: 10)** | |
| Predicted Feature | Sample of Annotated Features |
| ['Tylenol has not alleviated pain', 'albuterol last use 6 months ago']  ['tylenol did not help']  ['tylenol no relieve']  ['relieved by nothing']  ['tylenol no relief'] | ['albuterol inhaler with minor relief']  ['inhaler did not seem to help']  ['albuterol inhaler with minimal sympotmatic relief']  ['Tried inhaler with no relief'] |

**Table S4:** Examples of missing features that were difficult to extract due to semantic ambiguity or inconsistent phrasing.

| **Case 0: No-hair-changes-OR-no-nail-changes-OR-no-temperature-intolerance (Feature Number: 9)** | |
| --- | --- |
| Predicted Feature | Ground Truth Features |
| ['denies change in weight']  ['no skin changes']  ['denies hair changes']  ['no skin changes'] | ['Denies any sweating']  ['No sweating']  ['Denies heat intolerance', 'Denies cold intolerance']  ['no sweeling'] |
|  |  |
| **Case 4: Lack-of-other-thyroid-symptoms (Feature Number: 8)** | |
| Predicted Feature | Ground Truth Features |
| ['denies temperature intolerance']  ['denies hair loss']  ['denies palpitations']  ['denies heat/cold sensitivity']  ['no changes in skin']} | ['denies bowel chanegs', 'denies emperature intolerance']  ['denies any tremors', 'denies hair loss', 'denies heat intolerance', 'denies palpitations', 'denies changes in bowel habits']  ['denies any palpitations', 'denies heat/codl intolerance', 'denies changes in bowel habits']  ['Denies palpitation', 'Denies heat/cold sensitivity', 'Denies thinning of the hair', 'Denies changes in bowel movements']  ['No changes in skin', 'No changes in hair', 'No changes in bowel movements', 'No sweating', 'No palpitations'] |
